# Psychosocial Wellbeing among Patients with Breast Cancer during COVID-19

**DOI:** 10.3390/curroncol30040294

**Published:** 2023-03-30

**Authors:** Martine C. Maculaitis, Xianchen Liu, Alexandra Berk, Angelina Massa, Marisa C. Weiss, Samantha K. Kurosky, Benjamin Li, Lynn McRoy

**Affiliations:** 1 Cerner Enviza, Malvern, PA 19355, USA; 2 Pfizer Inc., New York, NY 10017, USA; 3 Invitae Corporation, San Francisco, CA 94103, USA; 4 Breastcancer.org, Ardmore, PA 19003, USA; 5 Lankenau Medical Center, Wynnewood, PA 19096, USA

**Keywords:** breast cancer, COVID-19, psychosocial wellbeing

## Abstract

The impact of coronavirus disease 2019 (COVID-19) on the wellbeing of breast cancer (BC) patients is not well understood. This study described psychosocial problems among these patients in the United States (US) during the COVID-19 pandemic. Data were collected from BC patients via an online self-report survey between 30 March–6 July 2021 to assess the prevalence of COVID-19 diagnosis history and potential depression, health-related quality of life, COVID-related stress, and financial toxicity. Patients with early-stage (eBC) and metastatic (mBC) disease were compared. Of 669 patients included in the analysis, the prevalence of COVID-19 diagnosis history (10.9% versus 7.7%) and potential depression (33.7% versus 28.3%) were higher in mBC than eBC patients. Patients with eBC (versus mBC) had higher scores on nearly all Functional Assessment of Cancer Therapy-Breast scales (all, *p* < 0.001). For the Psychological Impact of Cancer subscales measuring negative coping strategies, the emotional distress score was the highest (9.1 ± 1.8) in the overall sample. Comprehensive Score for Financial Toxicity scores were higher in eBC than in mBC patients (24.2 ± 11.3 vs. 21.3 ± 10.2, *p* < 0.001). Overall, the COVID-19-related stress score was highest for danger/contamination fears (8.2 ± 5.6). In conclusion, impairments to psychosocial wellbeing among patients during the pandemic were observed, particularly financial toxicity and poor mental health and emotional functioning, with greater problems among mBC patients.

## 1. Introduction

As of October 2022, over 624 million individuals have been diagnosed with coronavirus disease 2019 (COVID-19) globally, with over 6.5 million deaths [1]. There is a high risk of COVID-19 infection in cancer patients and cancer survivors owing to their immunosuppressive state compared to individuals without cancer, including increased vulnerability towards severe symptoms, poorer outcomes, and mortality [2,3,4,5].

COVID-19 has triggered dramatic disruptions or delays to services and care largely due to unprecedented strain on health systems and increased susceptibility to the physical and psychological effects of social isolation and economic upheaval [6,7]. Among those with cancer, there are serious concerns regarding infection risk, changes in treatment and downstream effects on cancer outcomes, and social determinants of health, which are more pronounced among patients with advanced cancer [6,7]. Furthermore, recent real-world data demonstrated an increase in cancer-related deaths due to the pandemic’s effect on health systems [8]. A recent study reported that, compared with pre-pandemic data, mortality associated with breast, colorectal, lung, and esophageal cancer is projected to increase by 7.9–9.6%, 15.3–16.6%, 4.8–5.3%, and 5.8–6.0%, respectively, in the next five years due to the postponement of cancer diagnosis [9].

Since breast cancer (BC) is the most common malignancy in women, with >2 million new cases and >650,000 deaths each year [10], there are concerns about patients with BC developing severe COVID-19 symptoms [11]. To date, the United States (US) has reported over 95 million COVID-19 cases and over 1 million deaths [1]. In addition to causing remarkable disruptions in the US healthcare system, the pandemic has also had a profound impact regarding delayed BC diagnosis and treatment [12]. Early in the pandemic, there were substantial declines in both screening and diagnostic mammography [13]. Moreover, treatment protocols for BC were modified, with patient-reported delays in treatment and limited chemotherapy administration [14,15].

Prior research suggests that patients with BC commonly experience psychological distress due to diagnosis and physical problems, sexual dysfunction, negative body image, and side effects of therapeutic procedures [16]. Additionally, a recent meta-analysis, which included 282,203 patients with BC, reported that depression and anxiety independently predicted all-cause mortality and cancer recurrence, respectively [17]. Rumination and worry also increase cancer-related distress among patients with BC, resulting in greater pain and more self-reported physical complaints [18]. With the COVID-19 pandemic imposing restrictions on public life and delays in medical services, negative impacts on physical, psychological, and cognitive functioning were reported among patients with BC [19,20]. Furthermore, a recent US-based mixed methods study reported that patients with BC experience psychosocial changes, such as anxiety, depression, and social isolation, in their attempt to reduce COVID-19 exposure [21]. Thus, COVID-19 may further exacerbate psychosocial problems attributed to BC, leading to more acute emotional distress and potentially increasing patient suffering.

Understanding how COVID-19 affects patients with BC, including clinical disease course, risk of severe COVID-19, and excess mortality, is essential for mitigation strategies, adaptation of site-specific models, and pathways of care [22]. Given the high prevalence of BC and the rapid transmission of COVID-19, it is important to understand the psychological impact of the COVID-19 pandemic on patients with BC, which may influence subsequent clinical outcomes. Despite the emergence of data on the clinical impact of COVID-19 on patients with cancer, limited research is available on psychosocial problems among those with BC during the COVID-19 pandemic [19,20,21,23,24,25,26,27,28]. Among those with BC, COVID-related anxiety is proportional to their risk of experiencing COVID-19 complications, such as being of older age, having comorbidities, and being at later stage of disease, suggesting that anxiety may be a suitable proxy for the degree of patients’ COVID-19 risk [29].

Being an international public health emergency, the COVID-19 pandemic has prompted an urgent need for the oncology community to derive a data-driven understanding of the complexities at the intersection of COVID-19 and cancer [30]. Furthermore, there is a paucity of studies reporting the impact of COVID-19 from the perspective of patients with BC.

The current analysis aimed to describe psychosocial wellbeing during the COVID-19 pandemic among US patients with BC. Specifically, this study assessed COVID-19-related stress, psychological coping with cancer, financial toxicity, and health-related quality of life (HRQoL) among a large, geographically diverse sample of patients to provide a more comprehensive understanding of the myriad psychosocial problems they may have experienced during the COVID-19 pandemic. Finally, this study sought to evaluate differences in psychosocial wellbeing by BC type (metastatic BC [mBC] and early-stage BC [eBC]), as well as to explore differences by COVID-19 diagnosis history (ever diagnosed and not ever diagnosed), which may help to identify subpopulations of patients with potential unmet needs for additional support.

## 2. Materials and Methods

### 2.1. Study Design and Data Source

This was an online self-report survey of patients with BC conducted between 30 March–6 July 2021. US-based patients were recruited from Invitae’s Ciitizen patient-mediated health records and real-world evidence platform and patient advocacy groups (METAvivor, TOUCH, The Breasties, SurvivingBreastCancer.org, and the Metaplastic Breast Cancer Global Alliance), with approximately 39% recruited from the Breastcancer.org patient community. A subset of survey participants was enrolled in Ciitizen (now part of Invitae Corporation, San Francisco, CA, USA). Ciitizen leverages the Health Insurance Portability and Accountability Act (HIPAA) right of access on patients’ behalf to collect and store their medical records and turns medical record documents into structured, longitudinal data that can be shared with whomever the patients want, for their own clinical treatment or for observational research and clinical trials. Key clinical and treatment data are extracted from the medical records to provide the patient with a visual summary of their treatment journey that can be used for second opinions or other personal care coordination needs. The extracted data are used for research purposes for patients who have consented to share their data for research use. In addition to receiving their own medical records, survey participants who enrolled in Ciitizen consented to share their de-identified data for the study. The current article focuses on the self-report survey results of participating patients with BC.

### 2.2. Study Sample

Eligible patients were aged ≥18 years, self-reported currently having a diagnosis of Stage 1, 2, or 3 or Stage 4 BC (eBC or mBC, respectively), and provided informed consent. Those who self-reported currently having Stage 0 BC or ductal carcinoma in situ (DCIS) were ineligible.

### 2.3. Study Measures

Demographics included age, race, US region, community type, household size, education, employment status, marital status, and income. Clinical/health characteristics included COVID-19 diagnosis history (ever diagnosed versus [vs.] not ever diagnosed), body mass index (BMI), BC type (eBC vs. mBC), BC stage at initial diagnosis, disease duration, diagnosed comorbidities, and current BC treatments; for the Charlson Comorbidity Index (CCI), weights are assigned to 11 health conditions and then summed; higher scores indicate greater comorbidity burden [31].

COVID-19-related stress was assessed using 3 subscales from the COVID Stress Scale (CSS), measuring perceived COVID-related danger/contamination fears, socioeconomic consequences, and traumatic stress (scores can range from 0–24; higher scores indicate greater COVID-related stress) [32]. Potential depression was measured with an 8-Item Patient Health Questionnaire (PHQ-8; scores range from 0–24, with scores ≥ 10 indicating potential depression) [33]. The 4 Psychological Impact of Cancer Scale (PIC) subscales represent different coping strategies (scores range from 3–12; higher scores indicate greater reliance on a given coping strategy) [34]. HRQoL was assessed via Functional Assessment of Cancer Therapy-Breast (FACT-B; 5 subscales and 2 total scores; score range varies by scale; higher scores indicate better HRQoL) [35]. The 11-item Comprehensive Score for Financial Toxicity (COST) assessed economic hardship (scores range from 0–44; lower scores indicate greater financial toxicity; scores of ≥26, ≥14–26, >0–14, and 0 indicate no, mild, moderate, and severe financial toxicity, respectively) [36,37]. Detailed information on the psychometric properties of psychosocial burden measures has been reported elsewhere [32,33,34,35,36,37].

### 2.4. Statistical Analyses

Analyses were performed using IBM SPSS^®^ version 28.0. Descriptive statistics were reported for demographics, clinical/health characteristics, and psychosocial wellbeing. This analysis included means and standard deviations (SDs) for continuous variables, with frequencies and percentages reported for categorical variables.

Comparisons by BC type and COVID-19 diagnosis history on FACT-B, PIC, COST, and CSS scores were performed using independent samples *t*-tests. Prevalence of COVID-19 diagnosis history and potential depression were reported as percentages with 95% confidence intervals (CIs). Spearman correlations (ρ) were conducted to examine the relationship between psychosocial wellbeing measures. Generalized linear models (GLMs), specifying negative binomial distribution and log link function, were used to examine the association between BC type and CSS subscales, adjusting for potential confounders. Based on a backward stepwise elimination procedure, GLM for CSS danger controlled for community type, potential depression, number of adults in the household, and income. GLM for CSS socioeconomic consequences controlled for education, current treatment with hormone therapy, community type, race, potential depression, current treatment with chemotherapy, and income. GLM for CSS traumatic stress controlled for potential depression, current treatment with targeted therapy, and income. Additionally, GLMs for all CSS scales controlled for age, BMI, CCI score, and COVID-19 diagnosis history due to conceptual relevance (i.e., COVID-19 risk factors). For correlations, *t*-tests, and GLMs, *p*-values < 0.05, 2-tailed, were considered statistically significant.

## 3. Results

### 3.1. Patient Characteristics

**Figure 1:**
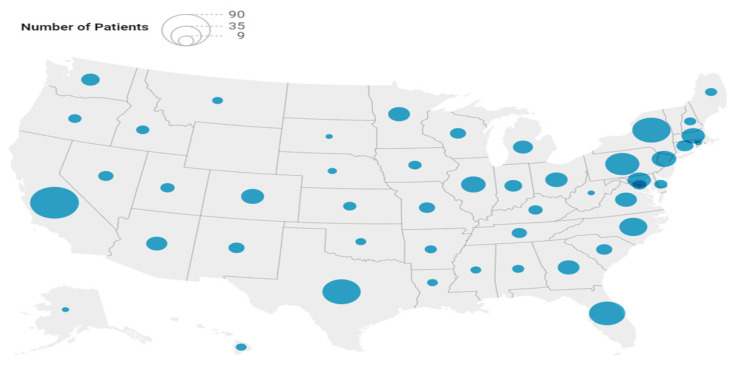
Number of Study Participants by US State. Note: Map created using the Datawrapper application (https://app.datawrapper.de (accessed on 2 March 2023)).

Overall, 669 patients with BC (aged 28–82 years) were included in the analyses. The number of patients who participated in the study by US state is depicted in Figure 1, with demographic and clinical/health characteristics of the study sample reported in Table 1.

Patients who participated in the study most often reported living in California (*n* = 92, 13.8%), New York (*n* = 55, 8.2%), Texas (*n* = 55, 8.2%), Florida (*n* = 49, 7.3%), or Pennsylvania (*n* = 43, 6.4%) (Figure 1). Participants had a mean age of 51.6 ± 11.3 years; most patients were female (99.0%) and White (83.9%) and had a college degree (69.5%) (Table 1). Roughly half had mBC at the time of the survey (51.4%). Over two-thirds (69.5%) had Stage 1, 2, or 3 BC at time of initial BC diagnosis. Depression (21.2%) and anxiety (17.9%) were the most frequently reported diagnosed comorbidities. At the time of the survey, patients were most often being treated with hormone therapy (58.7%), targeted therapy (38.4%), and chemotherapy (21.4%), with 11.4% not currently on any treatment.

### 3.2. Correlations between Psychosocial Wellbeing Measures

Most psychosocial wellbeing measures demonstrated adequate reliability, with Cronbach’s alpha values either approaching or exceeding 0.80 with the current sample. For PHQ-8 and COST, these values were 0.85 and 0.90, respectively, and on all three CSS subscales, Cronbach’s alpha was 0.92. These values were 0.74 and 0.75 for PIC cognitive distress and cognitive avoidance subscales, respectively. However, PIC emotional distress (0.68) and fighting spirit (0.46) subscales evidenced less internal consistency, possibly due, at least in part, to the small number of items in these subscales. As FACT-BC subscale scores are calculated slightly differently for males and females, we computed separate reliability estimates for this subscale and found values of 0.80 and 0.72 for males and females, respectively. For all other FACT-B metrics, Cronbach’s alpha values ranged from 0.77 (FACT-General) to 0.88 (FACT-Physical Wellbeing).

Supplementary Table S1 presents the correlations between psychosocial well-being measures in the study. Danger (ρ = 0.297) and traumatic stress (ρ = 0.385) CSS subscales and PIC cognitive distress (ρ = 0.382) and emotional distress (ρ = 0.385) subscales were positively correlated with PHQ-8 scores (*p* < 0.001). Stronger positive correlations were observed between FACT-B physical wellbeing subscale (ρ = 0.491), FACT-General total score (ρ = 0.529), and FACT-B total score (ρ = 0.553) with COST score (*p* < 0.001).

### 3.3. Prevalence of COVID-19 and Potential Depression

**Figure 2:**
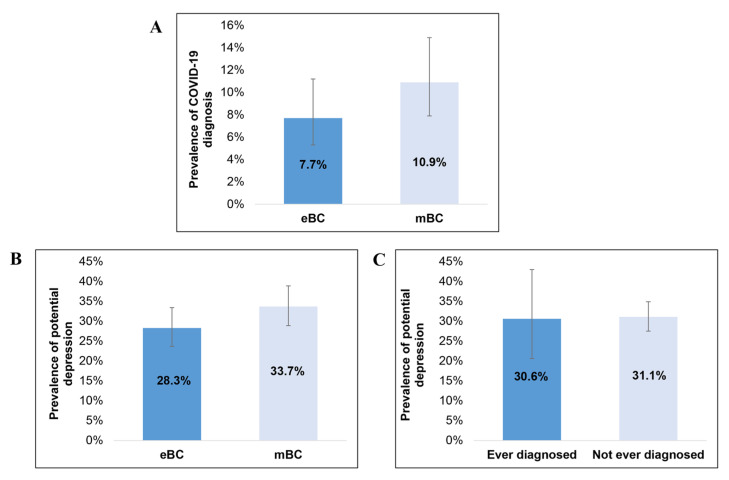
Prevalence of (**A**) COVID-19 diagnosis history by BC type, (**B**) potential depression by BC type, and (**C**) potential depression by COVID-19 diagnosis history. COVID-19, coronavirus disease 2019; eBC: early-stage breast cancer; mBC: metastatic breast cancer. Note: Error bars show 95% confidence intervals. A total of 8 patients were excluded from estimates of COVID-19 diagnosis history prevalence for BC type subgroups and from estimates of potential depression for COVID-19 diagnosis history subgroups (*n* = 7 responded “not sure” and *n* = 1 responded “prefer not to answer” on survey item about COVID-19 diagnosis history).

The overall prevalence of COVID-19 diagnosis history was 9.4% (95% CI: 7.4–11.8%). Patients with mBC (vs. eBC) had a higher prevalence of COVID-19 diagnosis history, although this difference was not statistically significant (10.9% vs. 7.7%, *p* = 0.157; Figure 2A).

The overall prevalence of potential depression was 31.1% (95% CI: 27.7–34.7%). The prevalence of potential depression was higher among patients with mBC (vs. eBC: 33.7% vs. 28.3%, *p* = 0.131; Figure 2B), but this difference did not reach statistical significance. Similar rates of potential depression were observed by COVID-19 diagnosis history (ever diagnosed: 30.6% vs. not ever diagnosed: 31.1%, *p* = 0.947; Figure 2C).

### 3.4. Psychosocial Wellbeing

The psychosocial wellbeing measures assessed in this study are presented in Table 2.

FACT-B subscale scores, from lowest to highest, were emotional (15.4 ± 4.8), functional (16.3 ± 6.1), social (17.4 ± 6.0), and physical (18.5 ± 6.3) wellbeing; mean BC subscale score was 22.8 ± 6.8. Higher scores were observed in patients with eBC (vs. mBC) on nearly all FACT-B subscales (*p* < 0.001). FACT-B scores did not differ by COVID-19 diagnosis history.

Among the three PIC subscales that measure negative coping strategies, scores were highest on emotional distress. Patients with mBC (vs. eBC) had higher scores on cognitive distress (5.6 ± 2.1 vs. 5.2 ± 1.7, *p* = 0.002) and cognitive avoidance (7.7 ± 2.3 vs. 6.9 ± 2.1, *p* < 0.001) subscales. PIC scores did not differ by COVID-19 diagnosis history.

The mean COST score for the overall sample was 22.7 ± 10.8. The mean COST score was higher for patients with eBC (vs. mBC; 24.2 ± 11.3 vs. 21.3 ± 10.2, *p* < 0.001). COST scores did not differ by COVID-19 diagnosis history.

### 3.5. COVID-19-Related Stress and Associated Factors

The highest CSS scores were observed for the danger (8.2 ± 5.6) subscale, followed by scores on socioeconomic consequences (3.7 ± 4.8) and traumatic stress (3.0 ± 4.2). CSS scores did not differ by BC type or COVID-19 diagnosis history (Table 2).

Parameter estimates from multivariable models are shown in Table 3. Multivariable analysis showed no significant association between BC type and CSS danger (*B* = −0.04, standard error [*SE*] = 0.09, *p* = 0.696), socioeconomic consequences (*B* = −0.09, *SE* = 0.10, *p* = 0.384), and traumatic stress (*B* = −0.12, *SE* = 0.13, *p* = 0.377) subscales. Having potential depression (*B* = 0.26, *SE* = 0.10) and lower income (*B* = 0.25, *SE* = 0.09) were associated with higher CSS danger scores (both, *p* < 0.01). Currently being treated with hormone therapy (*B* = 0.21, *SE* = 0.10) or chemotherapy (*B* = 0.25, *SE* = 0.12), being overweight/obese (*B* = 0.32, *SE* = 0.10), having potential depression (*B* = 0.46, *SE* = 0.10), and lower income (*B* = 0.30, *SE* = 0.10) were associated with higher CSS socioeconomic consequences scores (all, *p* < 0.05). Having a higher CCI score (*B* = 0.27, *SE* = 0.11), potential depression (*B* = 0.78, *SE* = 0.10), and lower income (*B* = 0.34, *SE* = 0.10) were associated with higher CSS traumatic stress scores (all, *p* < 0.05). Age was not associated with any CSS subscale scores.

**Figure 3:**
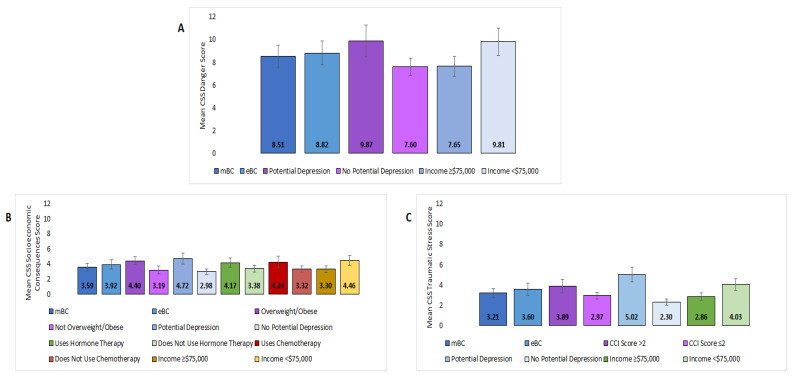
Adjusted means for BC type and variables significantly associated with (**A**) CSS danger score, (**B**) CSS socioeconomic consequences score, and (**C**) CSS traumatic stress score. CCI, Charlson Comorbidity Index; CSS, COVID Stress Scale; eBC, early-stage breast cancer; mBC, metastatic breast cancer. Note: Error bars show 95% confidence intervals.

Adjusted means by BC type and for those variables that demonstrated a statistically significant association with each CSS subscale are shown in Figure 3. For CSS danger subscale scores, differences between depression (potential depression [adjusted mean]: 9.87 vs. no potential depression: 7.60) and income (<$75,000: 9.81 vs. ≥$75,000: 7.65) groups were of similar magnitude. The largest mean differences in CSS socioeconomic subscale scores were observed among depression (potential depression: 4.72 vs. no potential depression: 2.98) and BMI (overweight/obese: 4.40 vs. not overweight/obese: 3.19) groups, with depression (potential depression: 5.02 vs. no potential depression: 2.30) and income (<$75,000: 4.03 vs. ≥$75,000: 2.86) groups exhibiting the largest mean differences in CSS traumatic stress subscale scores.

## 4. Discussion

### 4.1. Summary and Contributions

This study described psychosocial problems, including potential depression, COVID-19-related stress, psychological coping with cancer, HRQoL, and economic burden, in US patients with BC during the COVID-19 pandemic. Patients reported impairments to psychosocial wellbeing, specifically high rates of potential depression, poor HRQoL, emotional distress, and financial toxicity. Higher prevalence of COVID-19 diagnosis history and potential depression, lower HRQoL, and greater financial toxicity were found in patients with mBC than eBC.

Current treatment use in this study was generally comparable to a cross-sectional study assessing the psychosocial impact of BC during COVID-19, in which 74% received hormone or chemotherapy treatment prior to surgery [28]. However, only 11% of patients in our study had had surgery in the past 3 months. This could be attributed to disruptions in non-COVID-related health services during the pandemic [28]. Depression and anxiety are common psychiatric conditions among patients with BC [38,39], and they were likewise the most frequently reported comorbidities in this study.

The prevalence of COVID-19 diagnosis history was approximately 5.5 times higher than the self-reported positive test rate in the US general adult population [40]. However, data were collected for that study earlier in the pandemic than the current study (April–May 2020 vs. March–July 2021); thus, the prevalence differential may be smaller if considering equivalent time periods. The slightly higher prevalence of COVID-19 diagnosis history observed in mBC than eBC is similar to a prospective registry study that assessed the characteristics and outcomes of BC patients diagnosed with COVID-19, in which two-thirds of those with COVID-19 were treated for mBC, suggesting that COVID-19 was more prevalent in patients with mBC [41].

Patients with BC often suffer from chronic psychological stress caused by diagnosis or treatment-induced physical functioning changes, side effects, or impaired social functioning and HRQoL [16]. Furthermore, patients with BC are at increased risk of developing mental health conditions during the pandemic due to restrictions on daily life [20]. Our study findings support this proposition, as the prevalence of potential depression was higher in this study (31.1%), relative to a multinational systematic review and meta-analysis conducted pre-pandemic that reported a pooled prevalence of potential depression of 24.6% and 24.1% in the Americas region and high sociodemographic index countries, respectively [42]. Additionally, in the current study, the prevalence of potential depression among patients with BC was 4 times higher than the US general adult population [43]. The prevalence of potential depression was higher in mBC than eBC patients, consistent with prior research findings [38].

The scores on all FACT-B metrics in the present study were low, indicating poor HRQoL. Among all the FACT-B metrics assessed, scores on emotional and functional wellbeing were the lowest, suggesting the greatest HRQoL impairment in those domains. A survey of patients with eBC in seven countries, including the US, conducted just prior to the pandemic reported mean FACT-B and FACT-General total scores of 99.0 and 72.5, respectively, which was slightly higher than the FACT-B (94.3) and FACT-General (71.4) scores of patients with eBC in the current study [44]. Further, patients with mBC (vs. eBC) had lower scores on nearly all FACT-B metrics, suggesting worse HRQoL among those with mBC, which is consistent with another study that revealed lower HRQoL for mBC in each FACT-B domain [45].

Of the PIC subscales, scores were observed to be highest on emotional distress, indicating this was the most commonly employed negative strategy for coping with cancer. Furthermore, patients with mBC (vs. eBC) had higher PIC scores on cognitive distress and cognitive avoidance subscales. Our findings are similar to a previous cross-sectional study conducted during the pandemic, wherein patients with BC experienced more severe physical conditions, further worsening emotional distress and cognitive impairment from disruptions in medical services [20].

Additionally, economic concerns may further negatively impact psychosocial wellbeing, given financial challenges were widespread during the COVID-19 pandemic [46]. Our results echoed previous findings in which a majority of patients with cancer experienced financial toxicity [37], with the current study showing this impact being more pronounced among those with mBC. However, the low COVID-related stress observed in this study was similar to a recent study that demonstrated low CSS scores on danger, socioeconomic consequences, and traumatic stress subscales [19].

Patients with and without a COVID-19 diagnosis history were similar on all psychosocial wellbeing measures. This may be because we assessed COVID-19 diagnosis history, and diagnosis may not have been recent, whereas the longest recall period for psychosocial wellbeing measures was the previous 2 weeks. Additionally, it is possible that some patients tested positive for COVID-19 but were asymptomatic or had mild symptoms, thereby experiencing little impact on psychosocial wellbeing. Finally, COVID-19 diagnosis may not necessarily be the driver of poor psychosocial wellbeing; rather, it may be that general pressures of the pandemic, such as daily activity restrictions, healthcare disruptions, and social isolation, may lead to decrements in psychosocial wellbeing.

Overall, this study provides unique insights into the psychosocial problems experienced by US patients with BC during the COVID-19 pandemic and underscores the considerable unmet needs in this patient population. The collective findings suggest that BC imposes substantial psychosocial problems, particularly among those with mBC, with negative impacts observed across multiple domains. Our study results highlight the necessity for interventions that comprehensively enhance patient wellbeing and can help clinicians and public health policymakers identify specific areas in which patients may require further support.

### 4.2. Limitations and Strengths

This study’s results should be interpreted in light of relevant limitations. First, healthier patients and those interested in research may have been more likely to choose to participate in the study. Second, although our sample was distributed across all US regions, the first wave of the pandemic differentially impacted communities regarding the degree of restrictions imposed on healthcare and daily activities. Accordingly, patients living in communities with greater (vs. fewer) restrictions may have experienced larger decrements in psychosocial wellbeing. Third, self-reported health and clinical data are not independently verified and may potentially be subject to recall bias. Furthermore, given the study data are cross-sectional, causality cannot be inferred.

The inclusion of a broad array of validated psychosocial wellbeing measures, both general and BC-specific, is a notable strength of the current study. Consequently, the results are able to provide a more comprehensive assessment of the psychosocial problems experienced by patients with BC during the COVID-19 pandemic. Additionally, the large, geographically diverse sample is likewise an important strength of this study, which can enhance the external validity of the findings.

### 4.3. Future Work

Further research is warranted to ascertain whether temporal fluctuations in psychosocial wellbeing during the COVID-19 pandemic have occurred among patients with BC, as well as to examine longitudinal trends by US region. Moreover, given few patients with BC had a history of COVID-19 diagnosis, a future study will need to include a larger sample of these individuals to determine whether COVID-19 diagnosis has an incremental negative impact on psychosocial wellbeing above that which can be attributed to BC, as well as to gauge the magnitude of incremental impact. Similarly, it may also be fruitful to assess the extent to which COVID-19 severity and the presence of long-term sequelae following recovery from COVID-19 infection influence psychosocial wellbeing among patients with BC. An important next step for future research will be to evaluate the impact of psychosocial problems on key clinical outcomes, such as disease progression, survival, and response to treatment, and economic outcomes, including healthcare resource utilization, work productivity loss, and costs (direct and indirect), within the context of the COVID-19 pandemic. Such data may be vital for informing healthcare policymakers’ decisions regarding resource allocation and patient support in the event of future public health emergencies.

## 5. Conclusions

The current study provides important insights into psychosocial problems associated with BC during the COVID-19 pandemic in the US. COVID-19 diagnosis history was prevalent among patients with BC. Results also suggest impairments to psychosocial wellbeing regarding financial toxicity and poor mental health and emotional functioning, with greater psychosocial problems observed for those with mBC than eBC. Ultimately, these findings underscore the unique vulnerability, burden, and unmet needs among patients with BC, especially those with mBC, during the COVID-19 pandemic.

## Figures and Tables

**Table 1:** Sample Demographic and Health Characteristics.

Characteristics	*N* = 669
Age, mean (SD)	51.6 (11.3)
Sex, *n* (%)	
Male	7 (1.0)
Female	662 (99.0)
BMI Category *, *n* (%)	
Underweight	22 (3.3)
Normal weight	234 (35.6)
Overweight	179 (27.2)
Obese	223 (33.9)
Race, *n* (%)	
White	561 (83.9)
Black	60 (9.0)
Any other race	41 (6.1)
Prefer not to answer	7 (1.0)
Region of Residence, *n* (%)	
Northeast	156 (23.3)
Midwest	106 (15.8)
South	233 (34.8)
West	174 (26.0)
Community Type, *n* (%)	
Rural area	68 (10.2)
Small city or town	170 (25.4)
Suburb near a large city	297 (44.4)
Large city	134 (20.0)
Number of Adults in Household, mean (SD)	2.09 (0.89)
Number of Children in Household, mean (SD)	0.62 (1.02)
Education, *n* (%)	
Less than college degree	203 (30.3)
College degree or higher	465 (69.5)
Prefer not to answer	1 (0.1)
Marital Status, *n* (%)	
Not Married/Living with Partner	208 (31.1)
Married/Living with Partner	461 (68.9)
Employment, *n* (%)	
Not Employed	359 (53.7)
Employed	303 (45.3)
Prefer not to answer	7 (1.0)
Income, *n* (%)	
<$75k	275 (41.1)
$75k+	366 (54.7)
Prefer not to answer	28 (4.2)
Current BC Type, *n* (%)	
eBC	325 (48.6)
mBC	344 (51.4)
Stage at Initial Diagnosis, *n* (%)	
DCIS	36 (5.4)
Stage 0	11 (1.6)
Stage 1, 2, or 3	465 (69.5)
Stage 4	153 (22.9)
Don’t know	4 (0.6)
Disease Duration in Years, mean (SD)	5.50 (5.83)
Diagnosed Comorbidities ^†^, *n* (%)	
Depression	142 (21.2)
Anxiety	120 (17.9)
Another type of cancer, including leukemia/lymphoma	22 (3.3)
Chronic pulmonary disease	11 (1.6)
Mild liver disease	9 (1.3)
Kidney disease	5 (0.7)
Congestive heart failure	4 (0.6)
Chronic complications from diabetes	3 (0.4)
Moderate/severe liver disease	2 (0.3)
CCI score, mean (SD)	3.27 (2.40)
Current Treatments, *n* (%)	
Hormone therapy	393 (58.7)
Targeted therapy	257 (38.4)
Chemotherapy	143 (21.4)
Supportive/palliative care	90 (13.5)
Radiation	85 (12.7)
Surgery in past 3 months	74 (11.1)
Immunotherapy	23 (3.4)
Not receiving treatment	76 (11.4)

BC, breast cancer; DCIS, ductal carcinoma in situ; eBC, early-stage breast cancer; mBC, metastatic breast cancer; SD, standard deviation. * *N* = 11 patients were not included in the BMI calculation as some patients (*n* = 7) selected “prefer not to answer” on weight, and the remaining *n* = 4 had a BMI value that was an extreme outlier (defined as value ≥3 SDs above or below the mean for the overall patient sample). ^†^ Patients could select >1 response option. No patients (*n* = 0) reported having a diagnosis of Alzheimer’s disease/dementia, hemiplegia/paraplegia, or human immunodeficiency virus/acquired immunodeficiency syndrome (HIV/AIDS).

**Table 2:** Mean Scores of Psychosocial Wellbeing Measures (FACT-B, PIC, COST, CSS).

Scale, Mean (SD)		Overall (*N* = 669)	BC Type			COVID-19 Diagnosis History		
			eBC (*N* = 325)	mBC (*N* = 344)	*p*-Value	Ever Diagnosed (*N* = 62)	Not Ever Diagnosed (*N* = 599 *)	*p*-Value
FACT-B Scores	BC Subscale	22.8 (6.8)	23.0 (6.8)	22.7 (6.7)	0.534	22.0 (7.1)	23.0 (6.7)	0.277
	Physical Wellbeing	18.5 (6.3)	19.8 (6.0)	17.4 (6.4)	<0.001	19.1 (5.6)	18.5 (6.4)	0.478
	Social Wellbeing	17.4 (6.0)	18.2 (6.1)	16.6 (5.7)	<0.001	17.3 (5.8)	17.5 (5.9)	0.822
	Emotional Wellbeing	15.4 (4.8)	16.1 (4.6)	14.7 (4.9)	<0.001	15.2 (5.0)	15.4 (4.8)	0.830
	Functional Wellbeing	16.3 (6.1)	17.2 (6.0)	15.4 (6.0)	<0.001	16.2 (5.5)	16.3 (6.1)	0.888
	FACT-General	67.6 (17.9)	71.4 (17.5)	64.0 (17.6)	<0.001	67.8 (16.8)	67.6 (18.0)	0.943
	FACT-Breast Cancer	90.4 (22.5)	94.3 (22.3)	86.7 (22.1)	<0.001	89.8 (21.8)	90.6 (22.6)	0.788
PIC Scores	Cognitive Distress	5.4 (2.0)	5.2 (1.7)	5.6 (2.1)	0.002	5.2 (1.9)	5.4 (2.0)	0.412
	Cognitive Avoidance	7.3 (2.2)	6.9 (2.1)	7.7 (2.3)	<0.001	7.6 (1.7)	7.3 (2.3)	0.296
	Emotional Distress	9.1 (1.8)	9.0 (1.8)	9.2 (1.8)	0.192	9.0 (1.7)	9.1 (1.8)	0.630
	Fighting Spirit	10.0 (1.6)	10.0 (1.6)	10.0 (1.7)	0.916	10.1 (1.4)	10.0 (1.7)	0.546
COST Score	COST Total Score	22.7 (10.8)	24.2 (11.3)	21.3 (10.2)	<0.001	21.3 (9.8)	22.9 (10.9)	0.263
CSS Score	Danger	8.2 (5.6)	8.1 (5.6)	8.3 (5.6)	0.732	7.5 (6.0)	8.2 (5.6)	0.291
	Socioeconomic	3.7 (4.8)	3.5 (4.8)	3.9 (4.9)	0.339	4.6 (5.7)	3.6 (4.7)	0.122
	Traumatic Stress	3.0 (4.2)	3.0 (4.3)	2.9 (4.1)	0.662	2.8 (4.3)	3.0 (4.2)	0.684

BC, breast cancer; COST, Comprehensive Score for Financial Toxicity; COVID-19, coronavirus disease 2019; CSS, COVID Stress Scale; eBC, early-stage breast cancer; FACT-B, Functional Assessment of Cancer Therapy-Breast; mBC, metastatic breast cancer; PIC, Psychological Impact of Cancer Scale; SD, standard deviation. * *N* = 8 patients who selected prefer not to answer on COVID-19 diagnosis were not included in comparisons.

**Table 3:** Factors Associated with COVID-Related Stress: Generalized Linear Models.

	B	SE	95% LCL	95% UCL	*p*-Value
CSS Danger Scale					
Intercept	2.24	0.24	1.77	2.72	<0.001
mBC	−0.04	0.09	−0.21	0.14	0.696
Rural/small town	0.11	0.09	−0.06	0.29	0.205
Potential depression	0.26	0.10	0.07	0.45	0.006
Number of adults in household	0.06	0.05	−0.04	0.15	0.219
<$75,000 income	0.25	0.09	0.07	0.43	0.006
Age (years)	−0.01	0.00	−0.01	0.00	0.164
Overweight/obese BMI	0.00	0.09	−0.18	0.17	0.965
CCI score > 2	0.08	0.11	−0.13	0.28	0.462
Ever diagnosed with COVID-19	−0.14	0.15	−0.43	0.16	0.366
CSS Socioeconomic Scale					
Intercept	0.64	0.28	0.10	1.18	0.021
mBC	−0.09	0.10	−0.29	0.11	0.384
Less than college degree)	−0.17	0.11	−0.38	0.04	0.115
Age (years)	0.00	0.00	0.00	0.01	0.324
Currently using hormone therapy	0.21	0.10	0.02	0.41	0.034
Rural/small town	0.15	0.10	−0.05	0.34	0.141
White race	0.23	0.13	−0.02	0.48	0.066
Potential depression	0.46	0.10	0.26	0.66	<0.001
Overweight/obese BMI	0.32	0.10	0.12	0.52	0.001
Currently using chemotherapy	0.25	0.12	0.01	0.49	0.045
<$75,000 income	0.30	0.10	0.11	0.50	0.002
CCI score > 2	0.13	0.11	−0.09	0.35	0.248
Ever diagnosed with COVID-19	0.20	0.16	−0.11	0.51	0.211
CSS Traumatic Stress Scale					
Intercept	1.15	0.25	0.66	1.63	<0.001
mBC	−0.12	0.13	−0.37	0.14	0.377
CCI score > 2	0.27	0.11	0.05	0.50	0.018
Potential depression	0.78	0.10	0.58	0.98	<0.001
Currently using targeted therapy	−0.09	0.13	−0.34	0.16	0.488
<$75,000 income	0.34	0.10	0.15	0.53	<0.001
Age (years)	0.00	0.00	−0.01	0.00	0.335
Overweight/obese BMI	0.07	0.10	−0.13	0.27	0.497
Ever diagnosed with COVID-19	−0.15	0.17	−0.48	0.18	0.374

BMI, body mass index; CCI, Charlson Comorbidity Index; COVID-19, coronavirus disease 2019; CSS, COVID Stress Scale; eBC, early-stage breast cancer; LCL, lower confidence limit; mBC, metastatic breast cancer; SE, standard error; UCL, upper confidence limit.

## Data Availability

The study dataset is not publicly available due to the data collection only being granted exemption determination from an IRB for this specific protocol. The data presented in this study are available upon reasonable request from the corresponding author.

## Supplementary Materials

The following supporting information can be downloaded at: https://www.mdpi.com/article/10.3390/curroncol30040294/s1, Table S1: Intercorrelations Among Psychosocial Wellbeing Measures.

## Acronyms and Abbreviations

BC	Breast cancer
BMI	Body mass index
CCI	Charlson Comorbidity Index
CI	Confidence interval
COST	Comprehensive Score for Financial Toxicity
COVID-19	Coronavirus disease 2019
CSS	COVID Stress Scale
DCIS	Ductal carcinoma in situ
eBC	Early-stage breast cancer
FACT-B	Functional Assessment of Cancer Therapy-Breast
GLM	Generalized linear model
HIPAA	Health Insurance Portability and Accountability Act
HIV/AIDS	Human immunodeficiency virus/acquired immunodeficiency syndrome
HRQoL	Health-related quality of life
mBC	Metastatic breast cancer
PHQ-8	8-Item Patient Health Questionnaire
PIC	Psychological Impact of Cancer Scale
SD	Standard deviation
SE	Standard error
US	United States
vs.	Versus
