# Supplementary material for: Psychosocial Wellbeing among Patients with Breast Cancer during COVID-19
Source: Curr Oncol. 2023 Mar 30;30(4):3886–900. doi: 10.3390/curroncol30040294 (PMC10136618; doi:10.3390/curroncol30040294)
Supplement: Supplementary file 1 [file curroncol-30-00294-s001.zip › curroncol-2319122-supplementary.pdf]

SUPPLEMENTAL FILE

Table S1. Intercorrelations Among Psychosocial Wellbeing Measures.

N=669				Correlations															
				PHQ-8 Score	COST Total Score	CSS Scores			FACT-B Scores							PIC Score			
						Danger	Socioeconomic	Traumatic Stress	BC Subscale	Physical Well-Being	Social/Family Well-Being	Emotional Well-Being	Functional Well-Being	FACT-G	FACT-B	Cognitive Distress	Cognitive Avoidance	Emotional Distress	Fighting Spirit
Spearman's rho		PHQ-8 Score	Correlation Coefficient	—															
		COST Total Score	Correlation Coefficient	-0.465†	–														
	CSS Scores	Danger	Correlation Coefficient	0.297†	-0.242†	–													
		Socioeconomic	Correlation Coefficient	0.273†	-0.299†	0.512†	–												
		Traumatic Stress	Correlation Coefficient	0.385†	-0.281†	0.670†	0.493†												
	FACT-B Scores	BC Subscale	Correlation Coefficient	-0.588†	0.452†	-0.314†	-0.345†	-0.355†	–										
		Physical Well-Being	Correlation Coefficient	-0.625†	0.491†	-0.290†	-0.291†	-0.332†	0.584†	–									
		Social/Family Well-Being	Correlation Coefficient	-0.403†	0.322†	-0.224†	-0.199†	-0.231†	0.307†	0.309†	–								
		Emotional Well-Being	Correlation Coefficient	-0.578†	0.392†	-0.376†	-0.274†	-0.387†	0.493†	0.482†	0.342†	–							
		Functional Well-Being	Correlation Coefficient	-0.644†	0.420†	-0.278†	-0.266†	-0.333†	0.435†	0.574†	0.552†	0.504†	–						
		FACT-G	Correlation Coefficient	-0.723†	0.529†	-0.370†	-0.330†	-0.407†	0.585†	0.779†	0.708†	0.719†	0.852†	–					
		FACT-B	Correlation Coefficient	-0.752†	0.553†	-0.391†	-0.363†	-0.428†	0.759†	0.796†	0.653†	0.716†	0.803†	0.969†	–				
	PIC Scores	Cognitive Distress	Correlation Coefficient	0.382†	-0.289†	0.289†	0.286†	0.289†	-0.359†	-0.326†	-0.321†	-0.574†	-0.368†	-0.490†	-0.495†	–			
		Cognitive Avoidance	Correlation Coefficient	0.095*	-0.090*	0.093*	0.199†	0.123**	-0.101**	-0.076*	-0.059	-0.122**	-0.089*	-0.117**	-0.125**	0.235†	–		
		Emotional Distress	Correlation Coefficient	0.385†	-0.230†	0.321†	0.198†	0.328†	-0.340†	-0.314†	-0.159†	-0.685†	-0.304†	-0.441†	-0.451†	0.510†	0.174†	–	
		Fighting Spirit	Correlation Coefficient	0.069	-0.129**	0.133**	0.163†	0.137†	-0.140†	-0.131**	0.080*	-0.108**	-0.018	-0.062	-0.094*	0.083*	0.125**	0.304†	–

BC, breast cancer; COST, Comprehensive Score for Financial Toxicity; CSS, COVID Stress Scale; FACT, Functional Assessment of Cancer Therapy; FACT-B, Functional Assessment of Cancer Therapy – Breast; FACT-G, FACT, Functional Assessment of Cancer Therapy – General; PIC, Psychological Impact of Cancer; PHQ-8, 8-Item Patient Health Questionnaire.
*p<0.05; **p<0.01; †p<0.001.
